# Micro RNA in Semen/Urine from Non-Obstructive Azoospermia Patients as Biomarkers to Predict the Presence of Testicular Spermatozoa and Spermatogonia

**DOI:** 10.3390/life13030616

**Published:** 2023-02-23

**Authors:** Margo Willems, Céline Devriendt, Catharina Olsen, Ben Caljon, Toon Janssen, Inge Gies, Veerle Vloeberghs, Herman Tournaye, Dorien Van Saen, Ellen Goossens

**Affiliations:** 1 Biology of the Testis (BITE) Laboratory, Department of Reproduction, Genetics and Regenerative Medicine, Vrije Universiteit Brussel (VUB), Laarbeeklaan 103, 1090 Brussels, Belgium; 2 Brussels Interuniversity Genomics High Throughput Core (BRIGHTcore) Platform, Universitair Ziekenhuis Brussel (UZ Brussel), Vrije Universiteit Brussel (VUB), Laarbeeklaan 101, 1090 Brussels, Belgium; 3 Division of Pediatric Endocrinology, Department of Pediatrics, Universitair Ziekenhuis Brussel (UZ Brussel), Laarbeeklaan 101, 1090 Brussels, Belgium; 4 Brussels IVF, Universitair Ziekenhuis Brussel (UZ Brussel), Laarbeeklaan 101, 1090 Brussels, Belgium; 5 Department of Obstetrics, Gynecology, Perinatology and Reproduction, Institute of Professional Education, Ministry of Health of the Russian Federation, Sechenov First Moscow State Medical University, Trubetskaya Str. 8, b. 2, 119992 Moscow, Russia

**Keywords:** biomarkers, spermatogonia, non-obstructive azoospermia

## Abstract

About half of testicular sperm extraction (TESE) procedures in men with non-obstructive azoospermia (NOA), including men with Klinefelter syndrome (KS), are unsuccessful. To avoid unnecessary invasive surgery, biomarkers for spermatozoa were studied. In addition, markers for spermatogonia in testis tissue were explored. This study aimed to find biomarkers in the semen and/or urine of NOA patients to predict the presence of spermatogonia in the testis. Differentially expressed miRNAs were identified (1) between samples from patients with and without a positive TESE procedure as well as (2) between TESE-negative patients with and without spermatogonia. A total of thirteen upregulated miRNAs (ten in seminal plasma and three in urine) were found in the TESE-negative/spermatogonia-positive group compared to the TESE-negative/spermatogonia-negative group. These miRNAs could be potential biomarkers for spermatogonia; however, more research is necessary to validate their predictive power.

## 1. Introduction

About 12–15% of infertile men suffer from azoospermia. When the absence of spermatozoa in the ejaculate is due to the disturbed production of spermatozoa, which is the case in about 60% of azoospermic patients, it is referred to as non-obstructive azoospermia (NOA) [1]. The most frequent known genetic cause of NOA is Klinefelter syndrome (KS), a condition characterized by the presence of at least one supplementary X chromosome [2]. KS patients lose their germ cells at an early age, and during puberty, degeneration of the testicular tissue occurs. Yet, the testicular histology is patient-dependent, considering that tubular degeneration and fibrosis can be severe in some KS men, whereas others still have normal seminiferous tubules with ongoing spermatogenesis [3]. 

Currently, testicular sperm extraction (TESE) and, eventually, intracytoplasmic sperm injection (ICSI) are the fertility rescue techniques for adult NOA men [4]. For adult KS men, a microdissection TESE technique, which allows for selective biopsy, is the most common approach [5]. Nevertheless, no differences in retrieval rates have been shown between conventional and microdissection TESE [6]. Multiple TESE techniques are very invasive considering the current retrieval rates (±48%) of spermatozoa in unselected KS patients [7]. Fertility preservation strategies for KS boys have been considered, including the cryopreservation of testicular tissue containing spermatogonial stem cells (SSCs) for later use [8]. Nevertheless, options to restore the fertility through the collection of SSCs, e.g., in vitro maturation, are still experimental and controversial. Moreover, due to the poor chances of finding spermatogonia during childhood, it is assumed one should try to preserve the fertility of KS patients at early adulthood because spermatozoa retrieval rates were shown to be the highest at that time in some studies [9].

New approaches to predict whether a TESE procedure will be successful should be considered. A less invasive method to predict the presence of biomarkers for fertility may be to investigate seminal plasma or urine from NOA patients. MiRNAs were previously used as biomarkers for several diseases, such as cancer [10]. In relation to fertility, next-generation sequencing was utilized to identify thousands of microRNAs [11]. Differences in miRNA profiles between fertile and infertile males were studied, showing altered and defect-specific miRNA profiles in seminal plasma from infertile men with isolated asthenozoospermia, oligozoospermia, and teratozoospermia [12]. In addition, Von Kopylow et al. analyzed testis biopsies to identify spermatogonia-specific biomarkers that may be used to facilitate the isolation of SSCs [13]. 

Therefore, the aim of this study was to find differentially expressed miRNAs in both the seminal plasma and urine between NOA patients with a positive TESE procedure and those with a negative one (predictive biomarkers for the presence of testicular spermatozoa), as well as between NOA patients with a negative TESE who have spermatogonia and those who do not (predictive biomarkers for the presence of spermatogonia). With the presence of biomarkers in easily accessible fluids, the fertility strategy for KS patients, and in extension many NOA patients, could include (1) checking for biomarkers for spermatozoa, where, if the check is positive, a TESE procedure is performed; if the check is negative, (2) checking for spermatogonia-specific biomarkers such that a testicular biopsy can still be performed. If this check is positive, future in vitro spermatogenesis would be the aim once this technique becomes clinically available.

## 2. Materials and Methods

### 2.1. Study Design

First, miRNA was extracted from seminal plasma (*n* = 38) and urine (*n* = 26) samples from NOA patients. These samples were used to perform a sequencing study to find differentially expressed (*p*-value < 0.05 and |log2FC| > 2) miRNAs between NOA patients with and without a positive TESE. The miRNAs that were found to be upregulated in patients with a positive TESE could potentially be biomarkers for spermatozoa. Second, a differential miRNA expression study was performed on the seminal plasma and urine samples to compare the miRNAs between patients who had a negative TESE with and those without spermatogonia present in their testes. The upregulated miRNAs found in the samples from patients with spermatogonia could potentially be biomarkers for spermatogonia. Downstream analyses were performed to detect the target genes of the identified miRNAs as well as their association with the spermatogenesis process. 

### 2.2. Patient Samples

Semen and urine samples were collected at the andrology lab of the UZ Brussel from patients who had to undergo a TESE procedure. Samples were collected between June 2016 and March 2021. Seminal plasma samples from 38 adult patients were included in the study. Additionally, 26 urine samples were included, of which two were collected from KS boys (6 and 14 years old). An overview of the samples can be found in Supplementary Table S1. Liquefaction of the semen occurred by incubating the samples at 37 °C for 30 min. Subsequently, the samples were centrifuged at 1600× *g* for 10 min at room temperature (RT). The supernatant was collected and centrifuged at 11,900× *g* for 10 min. Urine samples were centrifuged at 1600× *g* for 10 min at RT. After collection, the samples were stored in Eppendorf tubes at −80 °C until use.

Next to semen and urine samples, testicular biopsy samples were collected from the patients during their TESE procedures. After arrival at the lab, testis tissue pieces were fixated in an alcohol–formalin acetic acid mixture (Q02022; International Medical Products, Oudergem, Belgium) for one hour and in TISSUE TEK-VIP (5990; Sakura, Berchem, Belgium) overnight, after which the tissue was embedded in paraffin. The paraffin-embedded samples were cut at different depths in sections 5 µm in thickness using a microtome (SM2010R; Leica Biosystems, Nußloch, Germany), after which they were used for immunohistochemistry. 

All obtained samples were included in this study after written informed consent. This study was approved by the internal review board (ethical committee) of the UZ Brussel (2015/121).

### 2.3. Immunohistochemistry

Paraffinized testicular tissue samples were stained for the presence of spermatogonia with the use of melanoma-associated antigen 4 (MAGE-A4). Samples were deparaffinized in xylene (3 × 10 min), rehydrated in decreasing alcohol concentrations (2 × 100%, 90%, and 70%), and rinsed with phosphate buffered saline (PBS; 10010023; Lifetechnologies, Gent, Belgium) for 5 min. Endogenous peroxidases were blocked with the use of hydrogen peroxide diluted in methanol for 30 min and washed with PBS for 5 min. Next, antigen retrieval was performed by heating the slides in a water bath for 95 min at 95 °C while immersed in citric acid. Slides were again washed in PBS for 5 min, and an additional blocking step was performed, using 4% normal goat serum (B304; Tebu Bio NV, Boechout, Belgium) diluted in PBS, for 30 min, after which the slides were incubated overnight at 4 °C with the primary antibody MAGE-A4 (provided by Dr Giulio Spagnoli, University of Basel, Switzerland) at a dilution rate of 1/200. Next, slides were washed (3 × 5 min), incubated with the secondary antibody (K5007—Dako REAL EnVision Detection System; Agilent Technologies, Heverlee, Belgium) for 60 min, and exposed to diaminobenzidine (1/50, provided with the Dako kit) for 1 min. Next, counterstaining (haematoxylin (5 min), hydrochloric acid (dip) and saturated lithium carbonated (dip)) and dehydration (increasing alcohol concentrations: 70%, 90%, and 2 × 100%) were performed, after which the slides were mounted with Entellan^®^ new (1079610100; Merck, Overijse, Belgium).

### 2.4. miRNA Extraction from Seminal Plasma and Urine

miRNA extraction from seminal plasma was performed using the Qiagen RNeasy Mini Kit (74104; Qiagen, Antwerp, Belgium). The protocol used was derived from the guidelines supplied with the kit; however, some changes were made. The samples were thawed and resuspended. First, 500 µL Trizol was added to 400 µL seminal plasma and incubated for 5 min at RT. Next, 150 µL chloroform (102445; Merck) was added, and the samples were incubated for 3 min at RT. The samples were centrifuged at 11,900× *g* for 15 min at 4 °C. The aqueous phase was transferred into a new tube, and one volume of 70% ethanol was added. Up to 700 µL of the samples was transferred to a RNeasy spin column in a 2 mL collection tube and centrifuged for 15 s at >8000× *g* at 4 °C. The flow-through was discarded, and the previous step was repeated until the whole sample was transferred to the spin column. Then, 700 µL buffer RWT was added to the spin column and centrifuged (15 s; >8000× *g*; 4 °C), after which the flow-through was discarded. A total of 500 µL RPE buffer was added in the same manner. Next, 500 µL 80% ethanol was added, and the samples were centrifuged for 2 min at >8000× *g* at 4 °C. The collection tubes with flow-through were discarded, and new 2 mL collection tubes were placed under the spin column. They were centrifuged with open lids at full speed for 5 min. Finally, the columns were placed on a 1.5 mL collection tube, 20 µL RNase-free water was added, and the samples were centrifuged for 1 min at >8000× *g* at 4 °C to elute the RNA. 

miRNA extraction from the urine samples was performed using the Norgen Urine miRNA Purification Kit (29000; Norgen Biotek Corp., Thorold, ON, Canada) according to the manufacturer’s instructions with a minor adaptation to the protocol (usage of 20 µL elution solution A instead of 50 µL). 

### 2.5. Data Analysis

The miRNA quantification of the samples was determined using the Qubit 4 Fluorometer (Q33238; Thermo Fisher Scientific, Gent, Belgium). Separation and quantitation measurements of the miRNAs were made with the Agilent 2100 Bioanalyzer system (G2939BA; Agilent technologies). Next, library preparation was performed as the first step of next-generation sequencing to allow the miRNAs to be identified. Finally, the sequencing was executed with the SMARTer^®^ smRNA-Seq Kit (635031; Takara Bio Inc., Kusatsu, Japan) for Illumina. 

The reads were mapped to the human genome (hg19), and these were then translated into a quantitative measure of miRNA expression using the miARma-Seq pipeline (version 1.7.5) [14]. The differential expression between the different groups was determined using the package edgeR. The pipeline runs fastQC for quality assessment, removes adapters with cutadapt, aligns the reads using Bowtie2, and finally counts the reads with featureCounts. A PCA analysis was run to check for outliers. Then, the significantly up- or downregulated miRNAs (*p*-value < 0.05 and |log_2_FC| > 2) were identified. Lastly, a novel database was used for the downstream analysis of the miRNAs, namely miRPathDB 2.0 [15]. In addition, targetscan was used to determine the target genes of the identified miRNAs [16].

The sequencing data were deposited in the GEO database (GSE224511).

## 3. Results

### 3.1. Characterization of Samples

Of the 38 seminal plasma samples which were included in the study, 30 were collected from patients with negative TESE results, whereas the other eight samples originated from patients with positive TESE results. Of the 26 urine samples, 23 originated from TESE-negative patients, and three were collected from TESE-positive patients. Within the seminal plasma samples of the TESE-negative patients, 20 samples were from patients lacking spermatogonia, whereas 10 samples were from patients with spermatogonia in their testicular biopsies. Within the urine samples of the TESE-negative patients, 17 samples were from patients lacking spermatogonia, whereas six samples were from patients with spermatogonia in their testicular biopsies (Supplementary Table S1).

### 3.2. Identification of Biomarkers for the Presence of Testicular Spermatozoa

First, differentially expressed miRNAs were identified between TESE-positive and TESE-negative patients. In the seminal plasma samples, a total of 624 miRNAs were identified, of which nine were significantly upregulated and six were significantly downregulated in the TESE-positive group. In the urine samples, a total of 492 miRNAs were detected, of which seven were significantly upregulated and six were significantly downregulated in the TESE-negative group (Table 1). 

When the downstream analysis was performed, the Kyoto encyclopedia of genes and genomes (KEGG) analysis showed the pathways in which the significant miRNAs contribute. 

**Figure 1:**
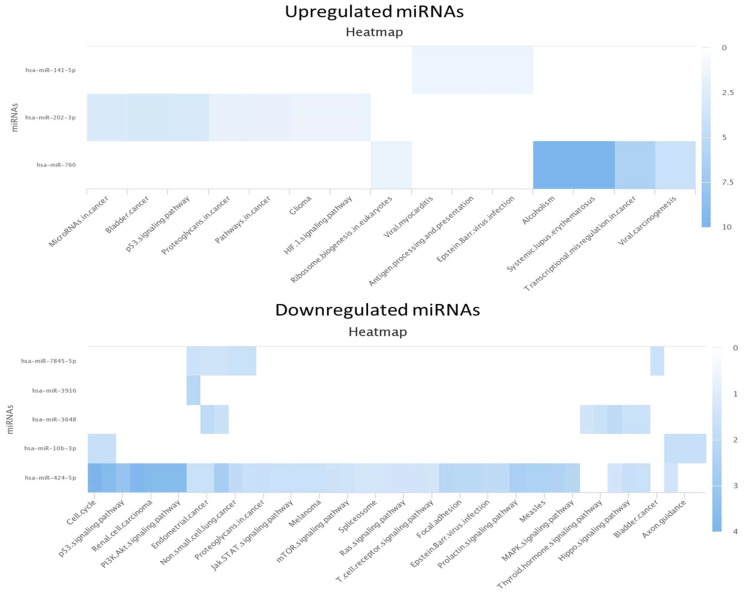
KEGG analysis for up- and downregulated miRNAs in seminal plasma.

**Figure 2:**
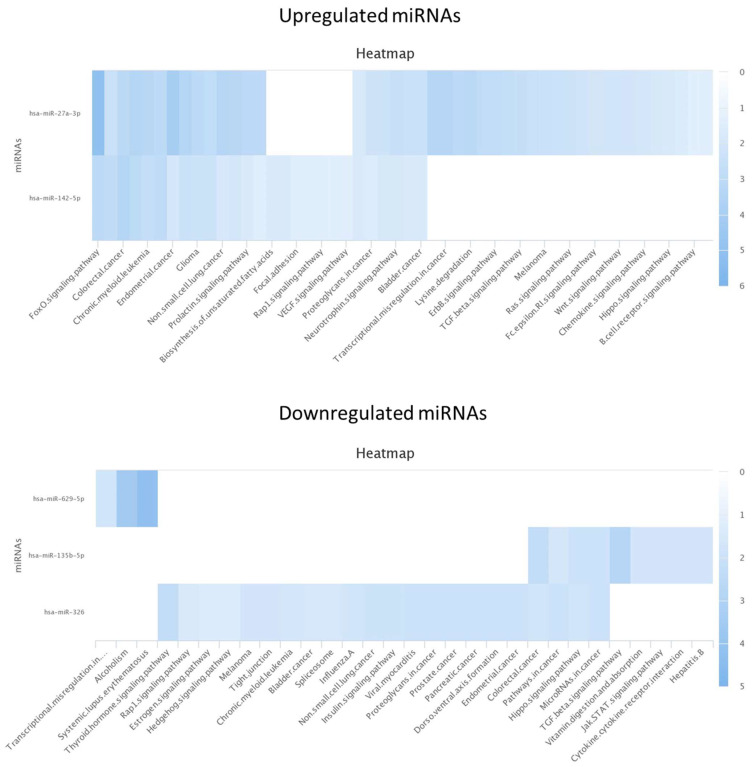
KEGG analysis for up- and downregulated miRNAs in urine.

The KEGG analysis for the miRNAs found in the seminal plasma showed only three of the miRNAs were upregulated, showing especially high upregulation of hsa-miR-760 in the pathways of alcoholism and systemic lupus erythematosus. For the downregulated miRNAs, only five out of eight miRNAs were identified in the database. hsa-miR-424-5p was found to play an especial role in several pathways including those involved in the cell cycle, P53 signaling pathway, and PI3K Akt signaling pathway (Figure 1). KEGG analysis was also performed for the urine samples, leading to the detection of the involved pathways of two miRNAs (hsa-miR-27a-3p and hsa-miR-142-5p), whereas the same analysis showed the involvement of different pathways of three out of six downregulated miRNAs (Figure 2). Unfortunately, no association between the identified miRNAs and pathways involved in spermatogenesis has yet been reported. When exploring other miRNAs that were identified but not significantly up- or downregulated, hsa-miR-494-3p was found to be downregulated in the TESE^−^ samples. 

### 3.3. Identification of Biomarkers for the Presence of Testicular Spermatogonia

In the second analysis, differentially expressed miRNAs were identified between MAGE-A4-positive and MAGE-A4-negative samples within the TESE-negative group to identify potential spermatogonia-specific biomarkers. In the seminal plasma samples, a total of 588 miRNAs were found, of which ten were significantly upregulated and four were significantly downregulated in the MAGE-A4-positive group. In the urine samples, a total of 476 miRNAs were detected, of which three were significantly upregulated and two were significantly downregulated in the MAGE-A4-positive group (Table 2). 

**Figure 3:**
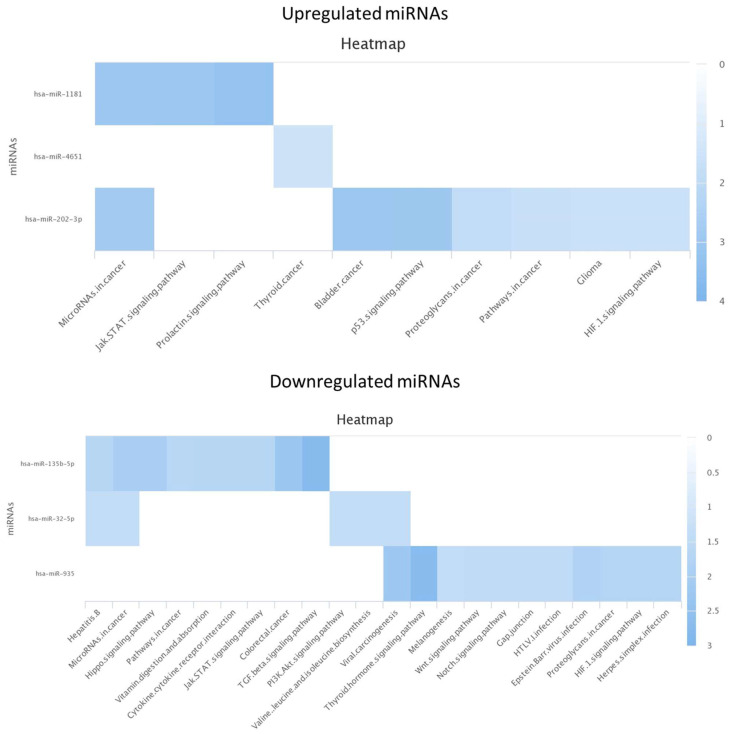
KEGG analysis for up- and downregulated miRNAs in seminal plasma (analysis 2).

When downstream KEGG analysis was performed, the pathways for the upregulated miRNAs found in the semen samples included, amongst others, the prolactin signaling pathway (for hsa-miR-1181), miRNAs in cancer (for hsa-miR-202-3p), and thyroid hormone signaling pathway (for hsa-miR-935). Additionally, for the downregulated miRNAs, different cancer-related pathways were found for three out of four identified miRNAs (Figure 3). For the up- and downregulated miRNAs found in the urine samples, the KEGG analysis showed no results. Again, the identified miRNAs have not yet been associated to germ cell development or to spermatogenesis pathways. Exploring other miRNAs that were identified, yet not significantly up- or downregulated, a few miRNAs appeared to contribute to the domain of interest. There is strong evidence that hsa-miR-365a-3p and hsa-miR-199a-3p, upregulated in the MAGE-A4-positive seminal plasma samples and urine, respectively, are associated with the ‘germ cell development’ pathway.

When exploring the target genes for the upregulated miRNAs, several sperm cell- and germ cell-related genes could be found, except for hsa-miR-3687. Most found target genes were related to infertility. An overview of the target genes for the upregulated miRNAs found in seminal plasma and urine is given in Table 3.

## 4. Discussion

A total of nine miRNAs were found to be significantly upregulated in seminal plasma, whereas seven upregulated miRNAs were found in the urine samples. These miRNAs could be potential biomarkers for testicular spermatozoa. Nevertheless, only two of the upregulated miRNAs were associated with the testis: hsa-miR-27a-3p and hsa-miR-202-3p. Overexpression of hsa-miR-202-3p was shown to be present in NOA patients, whereas hsa-miR-202-3p was shown to play a role in Sertoli-cell proliferation [17,18]. Ten miRNAs were shown to be upregulated in the seminal plasma and three in the urine of patients with a negative TESE procedure but with the presence of spermatogonia. These thirteen miRNAs could become potential biomarkers for the presence of spermatogonia. Nevertheless, no association was found between the significant miRNAs and germ cell development or spermatogenesis. Most of the miRNAs have already been studied in correlation with cancer. An overlap of three miRNAs were found between the first and second analyses: hsa-miR-202-3p (semen), hsa-miR-3653 (semen), and hsa-miR-4484 (urine).

Previous research on miRNAs related to sperm, spermatogenesis, or male infertility was performed. For instance, an abundant spermatozoal miRNA is hsa-miR-34c. This miRNA is highly conserved and was recently described to play a principal role in promoting the germinal phenotype during male gametogenesis [19]. MiR-371a-3p can be found in sperm-containing fluids. In ejaculates, there is a significant correlation between miRNA expression and sperm concentration and total sperm count, suggesting that the spermatozoa are potentially the source of miR-371a-3p production [20]. MiR-19b and let-7a are two miRNAs which are significantly increased in the seminal plasma of men with non-obstructive azoospermia compared to fertile controls. Thus, aberrant overexpression of these miRNAs could indicate spermatogenic failure [21]. A recent study investigated the expression profile of miRNAs in KS testes compared to controls. A total of 166 differentially expressed miRNAs were found [22]. Recently, miRNAs were investigated in seminal plasma, revealing hsa-miR-9-3p, hsa-miR-30b-5p, and hsa-miR-122-5p as potential biomarkers of male infertility and sperm quality [23]. 

Unfortunately, none of the miRNAs reported in these papers were found to be significantly up- or downregulated in our study. However, all three potential biomarkers reported by Joshi et al. (hsa-miR-9-3p, hsa-miR-30b-5p, and hsa-miR-122-5p) were found to be upregulated in both analyses of semen and urine samples [23]. 

## 5. Conclusions

In this study, a total of nine miRNAs (hsa-miR-892c-5p, hsa-miR-197-3p, hsa-miR-202-3p, hsa-miR-1224-5p, hsa-miR-3653, hsa-miR-760, hsa-miR-4525, hsa-miR-141-5p, and hsa-miR-365b-3p) were found to be upregulated in seminal plasma samples from non-azoospermic patients with positive TESE results compared to TESE-negative patients, whereas from the same patients, seven upregulated miRNAs (hsa-miR-4484, hsa-miR-142-5p, hsa-miR-27a-3p, hsa-miR-4488, hsa-miR-664b-3p, hsa-miR-30b-3p, and hsa-miR-5100) were found in urine samples. These miRNAs could potentially be biomarkers for spermatozoa. In addition, the differential miRNA expressions in semen and urine samples were investigated between samples with and without spermatogonia. This led to the identification of ten (hsa-miR-3687, hsa-miR-3195, hsa-miR-202-3p, hsa-miR-3656, hsa-miR-3653, hsa-miR-1225-3p, hsa-miR-6752-5p, hsa-miR-4651, hsa-miR-1181, and hsa-miR-513c-5p) and three (hsa-miR-4484, hsa-miR-5787, and hsa-miR-144-5p) miRNAs in semen and urine samples, respectively. However, the exact association between these miRNAs and fertility needs further investigation.

## Figures and Tables

**Table 1:** Differentially expressed miRNAs predicting the presence of testicular spermatozoa.

TESE^+^ vs. TESE^−^	
Seminal Plasma	
Significantly upregulated miRNA:	Significantly downregulated miRNA:
hsa-miR-892c-5p	hsa-miR-10b-3p
hsa-miR-197-3p	hsa-miR-3648
hsa-miR-202-3p	hsa-miR-424-5p
hsa-miR-1224-5p	hsa-miR-7846-3p
hsa-miR-3653	hsa-miR-3916
hsa-miR-760	hsa-miR-7845-5p
hsa-miR-4525	
hsa-miR-141-5p	
hsa-miR-365b-3p	
**Urine**	
Significantly upregulated miRNA:	Significantly downregulated miRNA:
hsa-miR-4484	hsa-miR-326
hsa-miR-142-5p	hsa-miR-135b-5p
hsa-miR-27a-3p	hsa-miR-183-3p
hsa-miR-4488	hsa-miR-4706
hsa-miR-664b-3p	hsa-miR-629-5p
hsa-miR-30b-3p	hsa-miR-4271
hsa-miR-5100	

**Table 2:** Differentially expressed miRNAs predicting the presence of testicular spermatogonia. The overlapping miRNAs between the first and second analyses are indicated in bold.

TESE^−^	
MAGE-A4^+^ vs. MAGE-A4^−^	
Seminal Plasma	
Significantly upregulated miRNA:	Significantly downregulated miRNA:
hsa-miR-3687	hsa-miR-32-5p
hsa-miR-3195	hsa-miR-935
**hsa-miR-202-3p**	hsa-miR-3605-3p
hsa-miR-3656	hsa-miR-135b-5p
**hsa-miR-3653**	
hsa-miR-1225-3p	
hsa-miR-6752-5p	
hsa-miR-4651	
hsa-miR-1181	
hsa-miR-513c-5p	
**Urine**	
Significantly upregulated miRNA:	Significantly downregulated miRNA:
**hsa-miR-4484**	hsa-miR-6821-3p
hsa-miR-5787	hsa-miR-106b-3p
hsa-miR-144-5p	

**Table 3:** Target genes for upregulated miRNAs.

Seminal Plasma	
MiRNA	Target Genes
hsa-miR-3195	SPEF1
hsa-miR-4651	SPATA33, SPATA2L, MOSPD3, SPATA6, STPG1, SPATA12, SPATA18, SPATA2, SPEM1, SPATA17, SPATA13, SPA17, SPATS2, SPATS2L, GSG1
hsa-miR-202-3p	SPATA7, SPATA18, DAZ3, DAZ2, DAZ4, DAZ1, STRBP, SPATA5, SPAG6, SPEM1, RSBN1L, GSG2
hsa-miR-3656	SPATS2L, GSG2
hsa-miR-1181	SPEF1
hsa-miR-3653	SPA17, BSPH1, CABS1, MAEL, SPATSL2, SPATA9, SPATA24, SPACA1, DAZL, SPATA12, ODFL2, NASP, SPATA5, SPAG9, RSBN1, RSBN1L, SMS, SPATA6L
hsa-miR-1225-3p	SPECC1L, SPATA2L, SPEF1, SPEM1, RSBN1L, SPATS2L, GSG2
hsa-miR-513c-5p	CABS1, TXNDC8, SPAGA17, RSBN1, DAZ4, DAZ2, DAZ3, DAZ1, MOSPD2, DAZL, EQTN, SPATA5, STPG2, ASUN, GMCL1, ODF2L, SPAG6, SPATA6, RSBN1L, TXNDC2, ODF2, STRBP, SPATS2, SPAG16, SPATS2L, SPATA6L, MAK
hsa-miR-6752-5p	SPAG11A, SPAG11B, SPAG7, CATSPER2, MOSPD3, SPACA4, SOHLH1, ODFL2, SPATA2L, STPG1, SPATA2, SPEM1, RSBN1L, SPAG9, SPATA6L
**Urine**	
**MiRNA**	**Target genes**
hsa-miR-4484	TXNDC2, SPATA13, SPECC1, RSBN1, SPATC1L, SPA17, SPATA5, GSG2
hsa-miR-5787	SPERT, STPG1, ODF1, MOSPD3, ODF4, DAZ3, DAZ4, DAZ1, DAZ2, ODF3L2, SPATA12, CATSPER4, SPATA13, SOHLH2, SPEM1, SPATA5, SPATA2L, ODF2L, SPECC1L, SPATA17, RSBN1L, GSS1
hsa-miR-144-5p	SPATA2, DAZ3, DAZ4, DAZL, DAZ2, DAZ1

## Data Availability

The sequencing data will be deposited in the GEO database upon review of the paper.

## Supplementary Materials

The following supporting information can be downloaded at: https://www.mdpi.com/article/10.3390/life13030616/s1, Table S1. Characterization of samples.
