# Supplementary material for: Micro RNA in Semen/Urine from Non-Obstructive Azoospermia Patients as Biomarkers to Predict the Presence of Testicular Spermatozoa and Spermatogonia
Source: Life (Basel). 2023 Feb 23;13(3):616. doi: 10.3390/life13030616 (PMC10051987; doi:10.3390/life13030616)
Supplement: Supplementary file 1 [file life-13-00616-s001.zip › life-2160434-supplementary.pdf]

Supplementary Table S1. Characterization of samples.

Sample	Age at Sampling	Karyotype	Seminal Plasma/Urine	Spermatozoa by TESE	Spermatogonia (MAGE-A4+)
1	31	46,XY	Seminal plasma	-	+
2	28	46,XY	Seminal plasma	-	+
3	39	46,XY	Seminal plasma	+	+
4	35	46,XY	Seminal plasma	+	+
5	25	46,XY	Seminal plasma	-	+
6	55	46,XY	Seminal plasma	-	+
7	29	46,XY	Seminal plasma	-	+
8	35	47,XXY	Seminal plasma	-	+
9	29	46,XY	Seminal plasma	-	+
10	35	46,XY	Seminal plasma	+	+
11	34	46,XY	Seminal plasma	-	-
12	42	47,XXY	Seminal plasma	-	-
13	37	46,XY	Seminal plasma	-	-
14	32	47,XXY	Seminal plasma	-	-
15	35	46,XY	Seminal plasma	+	+
16	51	46,XY	Seminal plasma	-	-
17	37	46,XY	Seminal plasma	-	-
18	26	46,XY AZF deletion	Seminal plasma	-	-
19	30	46,XY	Seminal plasma	+	+
20	35	46,XY	Seminal plasma	-	-
21	39	46,XY	Seminal plasma	+	+
22	29	47,XXY	Seminal plasma	-	-
23	49	46,XY	Seminal plasma	-	-
24	31	47,XXY	Seminal plasma	-	-
25	34	46,XY	Seminal plasma	-	-
26	28	46,XY	Seminal plasma	-	+
27	37	46,XY	Seminal plasma	+	+
28	45	46,XY AZF deletion	Seminal plasma	-	-
29	36	46,XY	Seminal plasma	-	-
30	36	46,XY	Seminal plasma	-	-
31	34	46,XY	Seminal plasma	-	+
32	31	46,XY	Seminal plasma	+	+
33	36	47,XXY	Seminal plasma	-	-
34	34	46,XY	Seminal plasma	-	+
35	37	46,XY	Seminal plasma	-	-
36	33	47,XXY	Seminal plasma	-	-
37	25	46,XY	Seminal plasma	-	-
38	27	47,XXY	Seminal plasma	-	-
39	31	46,XY	Urine	-	+
40	35	47,XXY	Urine	-	+
41	34	46,XY	Urine	-	-
42	42	47,XXY	Urine	-	-
43	33	46,XY	Urine	-	+
44	37	46,XY	Urine	-	-
45	51	46,XY	Urine	-	-

46	37	46,XY	Urine	-	-
47	26	46,XY	Urine	-	-
48	24	47,XXY	Urine	+	+
49	30	46,XY	Urine	+	+
50	35	46,XY	Urine	-	-
51	29	47,XXY	Urine	-	-
52	49	46,XY	Urine	-	-
53	31	47,XXY	Urine	-	-
54	34	46,XY	Urine	-	-
55	45	46,XY	Urine	-	-
56	36	46,XY	Urine	-	-
57	34	46,XY	Urine	-	+
58	31	46,XY	Urine	+	+
59	34	46,XY	Urine	-	+
60	37	46,XY	Urine	-	-
61	25	46,XY	Urine	-	-
62	27	47,XXY	Urine	-	-
63	14	47,XXY	Urine	-	-
64	6	47,XXY	Urine	-	+
